# Cytosolic Hsp70 as a biomarker to predict clinical outcome in patients with glioblastoma

**DOI:** 10.1371/journal.pone.0221502

**Published:** 2019-08-20

**Authors:** Friederike Lämmer, Claire Delbridge, Silvia Würstle, Frauke Neff, Bernhard Meyer, Jürgen Schlegel, Kerstin A. Kessel, Thomas E. Schmid, Daniela Schilling, Stephanie E. Combs

**Affiliations:** 1 Department of Radiation Oncology, Klinikum rechts der Isar, Technical University of Munich, Munich, Germany; 2 Institute of Pathology, Technical University of Munich, Munich, Germany; 3 Institute of Pathology, Klinikum Bogenhausen, Städtisches Klinikum München GmbH, Munich, Germany; 4 Department of Neurosurgery, Klinikum rechts der Isar, Technical University of Munich, Munich, Germany; 5 Institute of Radiation Medicine (IRM), Department of Radiation Sciences (DRS), Helmholtz Zentrum München, Neuherberg, Germany; 6 Deutsches Konsortium für Translationale Krebsforschung (dktk), Partner Site Munich, Munich, Germany; Universidad de Navarra, SPAIN

## Abstract

**Introduction:**

The major stress-inducible heat shock protein 70 (Hsp70) is induced after different stress stimuli. In tumors, elevated intracellular Hsp70 levels were associated on the one hand with radio- and chemotherapy resistance and on the other hand with a favorable outcome for patients. This study was undertaken to investigate cytosolic Hsp70 (cHsp70) as a potential biomarker for progression free (PFS) and overall survival (OS) in patients with primary glioblastomas (GBM).

**Methods:**

The cHsp70 expression in tumor tissue of 60 patients diagnosed with primary GBM was analyzed by immunohistochemistry. The cHsp70 expression was correlated to the PFS and OS of the patients.

**Results:**

A high cHsp70 expression was associated with a prolonged PFS (hazard ratio = 0.374, p = 0.001) and OS (hazard ratio = 0.416, p = 0.014) in GBM patients treated according to the standard Stupp protocol with surgery, radiotherapy and temozolomide.

**Conclusions:**

These data suggest that the intracellular Hsp70 expression might serve as a prognostic marker in patients with primary GBM.

## Introduction

Personalized treatment concepts are widely replacing established standard treatments for certain tumor types. The goal is individually tailored treatments with de-escalation, when appropriate, and intensification, when necessary. In primary glioblastoma (GBM), the current standard treatment regimen consists of neurosurgical resection followed by adjuvant radiation therapy (RT) and chemotherapy (CHT) with temozolomide (TMZ) [1]. Despite this multimodal treatment, the overall survival (OS) of this devastating disease is approximately 15 months [2]. The exact mechanisms for resistance of GBM to RT and CHT are not fully understood. Due to the genetic heterogeneity within the tumor including radiation-resistant tumor stem cells, there are several factors leading to therapy failure [3]. Therefore, the focus of many research groups worldwide is to improve the outcome of patients with GBM. During the last years, biomarkers became essential for a better prognosis and for an improved follow-up of RT. However, up to now, the O-6-methylguanin-DNA methyltransferase (MGMT) status is the only predictive marker for therapy response in GBM [4]. Patients with a methylation of the MGMT promoter benefit significantly more from the therapy with TMZ and show a better survival [1, 4]; moreover, this predictive marker not only reflects the outcome after TMZ, but also RT and other treatments, even if the underlying mechanisms are not fully explicable to date. Nevertheless, prognosis of GBM patients remains poor and biomarkers are desperately needed to improve therapy and prolong patient’s survival.

The major stress-inducible molecular chaperone heat shock protein 70 (Hsp70 or HSPA1A) exerts several housekeeping functions and protects cells against various forms of stress [5]. In normal cells cytosolic Hsp70 (cHsp70) is expressed at very low levels under physiological conditions, but it is strongly induced by a broad variety of stress stimuli (e.g. heat shock, oxidative stress, nutrient deprivation) [6]. In contrast to normal cells, tumor cells exhibit a high constitutive cHsp70 expression. Whereas cHsp70 contributes to tumor cell survival via multiple anti-apoptotic functions [7], extracellular Hsp70 in combination with pro-inflammatory cytokines such as interleukin-2 (IL-2) can elicit an anti-tumor immune response mediated by natural killer (NK) cells [8].

Although cHsp70 is well known to promote tumor cell survival, the association of cHsp70 expression and clinical outcome is contradictory. While in some tumor entities a high cHsp70 expression is associated with bad prognosis, in others, it is associated with a more favorable prognosis [9–15]. Therefore, in the present work, we aim to evaluate the correlation of cHsp70 expression with clinical outcome in primary GBM.

## Materials and methods

### Patients

From our institutional database, 60 patients with primary GBM treated with surgery, RT and TMZ of which tumor specimens were available, were analyzed. This retrospective study was approved by the local Ethics Committee (5625–12) of the Klinikum rechts der Isar (TUM) on 17 Dec 2012 and was conducted in accordance with the ethical standards of the 1964 Declaration of Helsinki and its later amendments. Informed consent was waived by the local Ethics Committee due to the retrospective study design.

Patients’ characteristics are shown in Table 1. All patients underwent gross total resection and were treated postoperatively with RT consisting of photons and concomitant TMZ treatment (median 6 cycles), according to the standard regimen published by Stupp et al. [1]. The MGMT methylation status of the GBM patients was evaluated with MethylQESD [16]. MGMT unmethylated are patients with less than 8% MGMT promoter methylation, whereas MGMT methylated are patients with more than 8% MGMT promoter methylation [17]. All patients were included in a tight follow-up program including clinical as well as imaging-based follow-up in an interdisciplinary setting of radiation oncology, neurology and neurosurgery. The median follow-up time was 16 months (range 1–76 months). Tumor recurrence was evaluated by radiologists based on MR images. In uncertain cases, the decision was made by an interdisciplinary tumor board.

**Table 1:** Demographic characteristics of the GBM patients.

	Patients			
	All	All (%)	cHsp70 low	cHsp70 high
All	60	100	22	38
**Sex (No.)**				
Male	35	58	12	23
Female	25	42	10	15
**Age (years)**				
Median	58		55.5	58.5
Range	20–78		41–70	20–78
**MGMT-Status (No.)**				
unmethylated (< 8%)	37	62	19	18
methylated (≥ 8%)	23	38	3	20
**PFS (months)**				
Median	12.5		9.7	15.6
Range	0.7–52.4		0.7–22.0	1.2–52.4
**OS (months)**				
Median	16.4		15.5	18.4
Range	0.7–76.1		0.7–27.1	1.2–76.1

MGMT: O-6-methylguanin-DNA methyltransferase, PFS: progression free survival, OS: overall survival.

### Immunohistochemistry

All 60 primary GBM patients (WHO grade IV) [18] were diagnosed based on haematoxylin/ eosin staining and the isocitrate dehydrogenase (IDH1) wild type status, which was determined by immunohistochemistry. All diagnoses were performed by an experienced neuropathology team. Tissue samples for diagnostic, molecular genetic analysis, and the specimens for this retrospective study were collected at the Klinikum rechts der Isar (TUM), and at the Klinikum Bogenhausen (STKM), both in Munich, Germany. The formalin fixed paraffin embedded (FFPE) sections of the neurosurgical specimen were analyzed by immunohistochemistry for Hsp70 (cmHSP70.1, IgG1, multimmune GmbH, Munich, Germany). Briefly: 2 μm sections were deparaffinised followed by epitope unmasking in citric acid based buffer (pH 6.0). The endogenous peroxidase was quenched with 3% H_2_O_2_. Slides were blocked with DAKO Antibody Diluent with added serum. Each slide was incubated with the Hsp70 antibody diluted in Antibody Diluent overnight at 4 °C followed by incubation with the HRP-linked secondary anti-mouse antibody and was detected with ImmPACT DAB chromogen. Counterstaining was performed with haematoxylin.

### Evaluation of immunohistochemistry and statistical correlation with clinical outcome

The staining of cHsp70 was analyzed semi-quantitatively in the tumor area. Regarding the cytoplasmic staining intensity of cHsp70, two categories of cHsp70 expression were defined: cHsp70 low consisted of samples with 10% or fewer positive cells, while cHsp70 high consisted of more than 10% positive cells. 10% was used as a cut off to exclude false positive staining of non-tumor cells like macrophages, microglia and reactive astrocytes. Scoring was done blindly without knowledge of patients’ data, by independent experienced neuropathologists. The cHsp70 expression was correlated with patients’ PFS and OS using the Kaplan-Meier survival analysis and the Cox regression analysis (R 3.2.2, package survival 2.38–3). A p-value ≤ 0.05 was considered significant.

## Results

**Fig 1:**
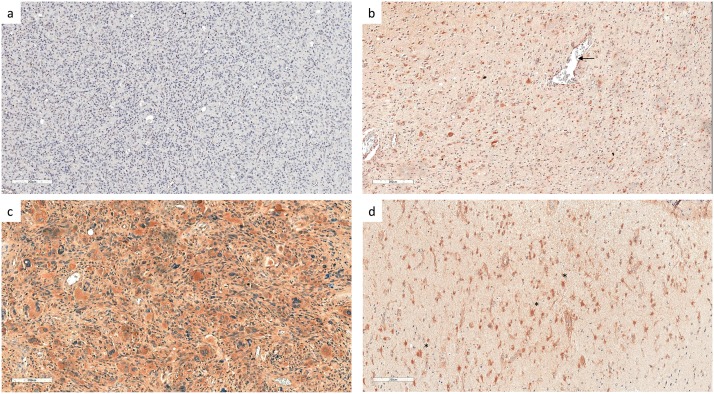
cHsp70 Immunohistochemistry in GBM tumors. Scale bar = 200 μm. (a) cHsp70 low expressing tumor. (b) Tumor with moderate cHsp70 staining. The arrow indicates Hsp70 expressing vascular endothelium. (c) cHsp70 high expressing tumor. (d) Tumor infiltration zone: distinction between cHsp70 expressing tumor cells and cHsp70 expressing reactive astrocytes cannot be made with absolute certainty. Examples for questionable cells are indicated by asterisks.

To evaluate the clinical significance of cHsp70 in primary GBM (IDH1-wildtype), tissue sections of 60 patients were analyzed. The cHsp70 expression was higher in the tumor cells than in the surrounding normal tissue, but showed high variations, with a disseminated staining pattern within the tumor (Fig 1a and 1b). Macrophages and the endothelium of vessels reacted also positively with the cHsp70 antibody (Fig 1b). Moreover, some cHsp70 positive cells matched the histological criteria of reactive astrocytes: branchy, cellular hypertrophy, eccentric enlarged nuclei, and end-feet termination on blood vessels [19] (Fig 1d). GBM are highly infiltrated by macrophages and microglia, therefore, to exclude false positive staining, 10% was used as a cut off level between low and high expressing tumors. While 38 (63.3%) of all GBM showed a high cHsp70 expression (Fig 1b and 1c), 22 (36.6%) were scored as Hsp70 low (Fig 1a).

**Fig 2:**
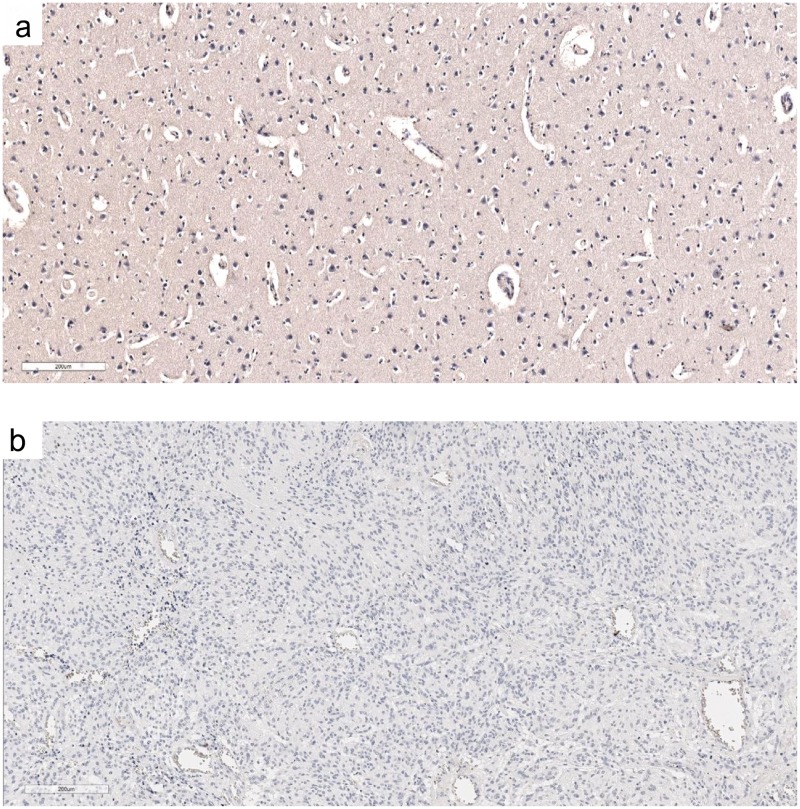
Immunohistochemistry. Scale bar = 200 μm. (a) normal brain stained with cmHSP70.1 antibody. (b) GBM stained with IgG control antibody.

The two controls, healthy brain tissue stained with antibody against cHsp70 and GBM stained with isotype control antibody, showed no positive staining (Fig 2).

**Fig 3:**
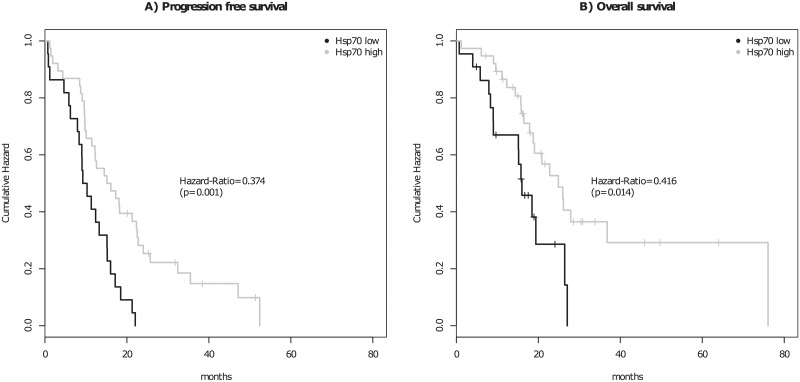
Kaplan-Meier survival analysis of progression free survival (a) and overall survival (b) of analysed patients, according to cHsp70 expression. Low Hsp70 expression: up to 10% positive tumor cells; high Hsp70 expression: more than 10% positive tumor cells.

The PFS of patients with low cHsp70 expressing tumors (median = 9.7 months) was 0.374 times (p = 0.001) shorter than of patients with high expressing tumors (median = 15.6 months) (Fig 3a). These results are comparable to the OS of these patients; GBM patients with a high cHsp70 expressing tumor had a median OS of 18.4 months, whereas the median OS of patients with low cHsp70 expressing tumors was 15.5 months (Fig 3b). The adjusted Hazard-Ratio for mortality associated with a high cHsp70 expression was 0.416 (p = 0.014).

**Fig 4:**
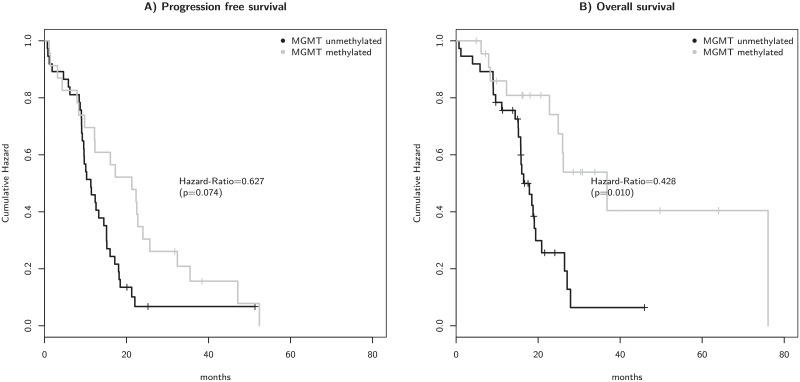
Kaplan-Meier survival analysis of progression free survival (a) and overall survival (b) of analysed patients separated by the MGMT status.

The MGMT promoter methylation status is well known to impact OS [4, 20]. Therefore, patients were further analyzed according to their MGMT promoter methylation status. As expected, patients with MGMT promoter methylation (n = 23) survived longer (median 36.8 months) than those with tumors containing an unmethylated promoter (n = 37, median 16.5 months) (Hazard-Ratio = 0.428, p = 0.003) (Fig 4). In line with the literature, PFS was not significantly different between patients with methylated MGMT promoter and unmethylated MGMT promoter [20].

Multivariate analysis of MGMT promoter methylation status and Hsp70 expression revealed no significance for PFS (Table 2). In contrast, MGMT promoter methylation status was significant concerning the OS.

**Table 2:** Univariate and multivariate analysis of progression-free survival (PFS) and overall survival (OS).

	PFS				OS			
	univariate		multivariate		univariate		multivariate	
	p-value	Exp(B)	p-value	Exp(B)	p-value	Exp(B)	p-value	Exp(B)
Hsp70	0.001	0.374	0.060	1.940	0.014	0.416	0.332	1.468
MGMT	0.074	0.627	0.277	0.671	0.010	0.428	0.027	0.367

Hsp70: Heat shock protein 70, MGMT: O-6-methylguanin-DNA methyltransferase, PFS: progression free survival, OS: overall survival

**Fig 5:**
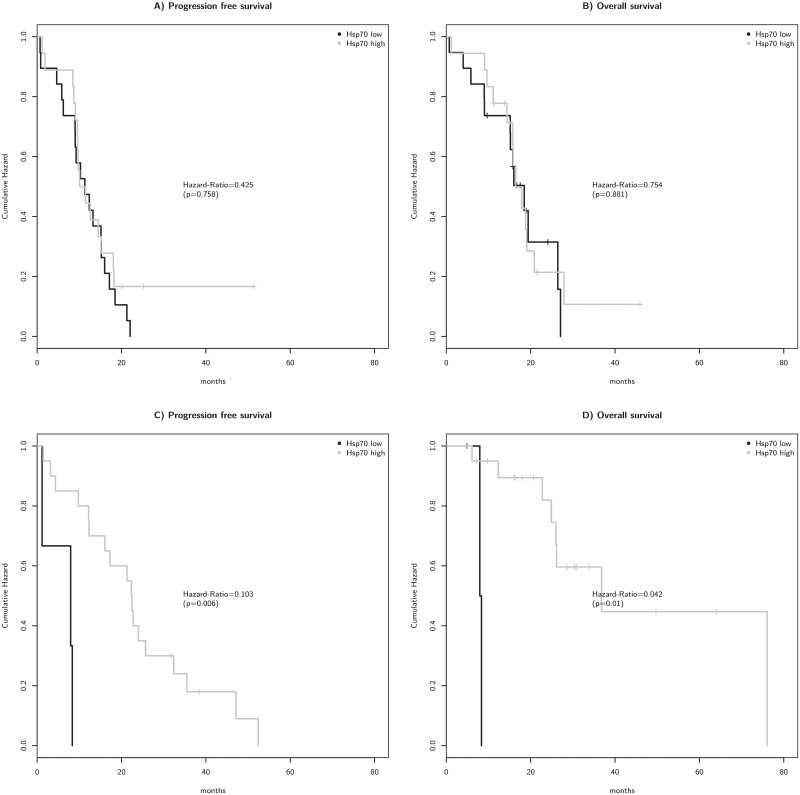
Kaplan-Meier survival analysis of progression free survival (a) and overall survival (b) of 37 MGMT unmethylated GBM patients and progression free survival (c) and overall survival (d) of 23 MGMT methylated GBM patients, according to cHsp70 expression. Low Hsp70 expression: up to 10% positive tumor cells; high Hsp70 expression: more than 10% positive tumor cells.

In the unmethylated MGMT group, there was no significant difference in the PFS and OS between patients with low and high cHsp70 expressing tumors (Fig 5a and 5b). However, within the MGMT methylated group patients with cHsp70 high expressing tumors have a better PFS (Hazard-Ratio = 0.103, p = 0.006) and OS (Hazard-Ratio = 0.042, p = 0.01) (Fig 5c and 5d). As the group of methylated patients with Hsp70 low expressing tumors was very small, this trend has to be validated in a larger cohort.

## Discussion

Overexpression of the anti-apoptotic molecular chaperone Hsp70 has been observed in many tumor entities [7] and a positive correlation with the WHO tumor grade has been reported for glioma [21, 22]. However, the prognostic relevance of Hsp70 expression seems to be cancer specific. For breast cancer [9, 23], head and neck cancer [10, 15], non-small cell lung cancer [14], and malignant melanoma [24] a high cHsp70 expression in tumors correlates with better survival of the patients. On the other hand, in hepatocellular carcinoma [25], intestinal cancer [11], or colon cancer [26], high cHsp70 expression in tumors is associated with a poor prognosis. The objective of this study was to analyze the cHsp70 expression in human primary GBM as a potential biomarker to predict the outcome of therapy.

As IDH1-wildtype (primary) and mutant (secondary) GBM have a different pathogenesis and a different outcome, in the present study only primary GBM were investigated as they reflect a homogeneous histological group of patients. In line with the literature, the patient collective comprises 62% MGMT unmethylated GBM and 38% methylated GBM [4].

Our study demonstrated that PFS and OS was significantly better in GBM patients with high cHsp70 expression levels. In contrast to our data, Hermisson et al. [27] did not find a significant difference in PFS of GBM patients, when separated according to their cHsp70 expression status. However, in their study GBM patients were treated with various regimens and not according to the Stupp protocol [1]. Furthermore, these patients were not stratified according to their IDH1 and MGMT status.

A very recent study investigated the Hsp70 expression in primary (n = 24) and secondary (n = 16) GBM patients [19]. They did not find any difference in PFS and OS of GBM patients stratified by their Hsp70 expression, detected by immunohistochemistry. However, they included and compared with each other primary and secondary GBM patients, which have a very different outcome; moreover, the heterogeneity in patient subpopulations weighs even stronger due to the small patient number included. Unfortunately, they do not provide any information about patients’ therapy.

Muth et al. [28] differentiated between intra- and extracellular Hsp70 levels in primary and recurrent GBM samples. Whereas intracellular Hsp70 levels have been found to be equally expressed in primary GBM tumors and relapse tissue, extracellular Hsp70 levels were found to be significantly enhanced only in relapse tissue. In this study, increased extracellular Hsp70 levels have been found to be correlated with an improved OS. However, data was collected only from 9 patients treated with heterogeneous therapy regimens.

In contrast to these data, our present results indicate a prognostic value of cHsp70 expression in primary GBM patients who are uniformly treated according to the Stupp protocol.

As it is known that Hsp70 can be actively released by tumor cells into the extracellular milieu [29], we hypothesize that GBM with high cHsp70 levels might secrete Hsp70 into the extracellular milieu. Extracellular Hsp70, in combination with pro-inflammatory cytokines such as IL-2, has been demonstrated to stimulate NK cells [30–32]. We hypothesize that this inflammatory milieu could be generated by an immunogenic cell death, which could be induced by RT in combination with TMZ. Therefore, we assume that the favorable effects of high cHsp70 levels in GBM tumors might be attributed to an increased anti-cancer immune response mediated by in vivo activated NK cells [33, 34]. The immunostimulatory capacity of Hsp70 in combination with the proinflammatory cytokine IL-2 has been confirmed in a phase I trial and is presently being tested in an ongoing phase II clinical trial. These studies showed that colon and lung cancer patients can generate antitumor immune responses after stimulation of NK cells with IL-2 and an Hsp70-derived peptide [30, 32, 35].

## Conclusions

In conclusion, our study demonstrates that the cHsp70 expression within the tumor region might provide a novel biomarker for PFS and OS in GBM patients. Further prospective trials will have to confirm the clinical value of cHsp70 in combination with other classical biomarkers (e.g. MGMT) in GBM.
