# Correction: Cytosolic Hsp70 as a biomarker to predict clinical outcome in patients with glioblastoma

**DOI:** 10.1371/journal.pone.0248612

**Published:** 2021-03-11

**Authors:** Friederike Lämmer, Claire Delbridge, Silvia Würstle, Frauke Neff, Bernhard Meyer, Jürgen Schlegel, Kerstin A. Kessel, Thomas E. Schmid, Daniela Schilling, Stephanie E. Combs

The raw data underlying Figs 3 and 4 are missing from the list of Supporting Information. The authors have provided the data as Supporting Information file S1 Dataset. With this correction [1], all relevant data are now provided.

The Data Availability statement for this paper is incorrect. The correct statement is: All relevant data are within the paper and its Supporting Information files.

## Supporting information

S1 Dataset Clinical data of individual patients. (XLSX)
